# Sustainable Mix Design of Sugarcane Bagasse Ash Concrete via AutoML-Assisted Multi-Objective Optimization

**DOI:** 10.3390/ma19173704

**Published:** 2026-08-31

**Authors:** Yang Cui, Zhengyu Fei, Yi Zhao, Bo Yang, Shixue Liang

**Affiliations:** 1 Zhejiang Hualin Construction Group Co., Ltd., Hangzhou 310000, China; cuiyanggwj@163.com; 2 School of Civil Engineering and Architecture, Zhejiang Sci-Tech University, Hangzhou 310018, China; fzy01060020@163.com (Z.F.); zhaoyi7368@163.com (Y.Z.); youngbo@zstu.edu.cn (B.Y.); 3 School of Construction, Zhejiang College of Construction, Hangzhou 310000, China

**Keywords:** sugarcane bagasse ash (SCBA), mix design, multi-objective optimization, automated machine learning (Auto-ML), NSGA-III, CO_2_ emission

## Abstract

**Highlights:**

- Sugarcane bagasse ash (SCBA) concrete mix design is optimized for low-carbon use.
- Automated machine learning predicts compressive strength from the mix parameters of SCBA concrete.
- NSGA-III derives optimal SCBA concrete mix designs considering strength, cost, and CO_2_ emissions.
- Compression tests confirm the reliability of the optimized mix designs of SCBA concrete.

**Abstract:**

The environmental impact of cement production has become a growing global concern due to its substantial contribution to CO_2_ emissions. Sugarcane bagasse ash (SCBA), as a supplementary cementitious material, offers a sustainable alternative by partially replacing cement and reducing the carbon footprint of concrete. However, determining optimal mix proportions that balance mechanical strength, cost efficiency, and environmental benefits remains a complex challenge. In this study, a multi-objective optimization framework was developed by integrating automated machine learning (Auto-ML) with the NSGA-III algorithm. A surrogate model for predicting compressive strength was constructed using the TPOT-based Auto-ML tool, achieving high predictive accuracy with R^2^ values of 0.993 and 0.908 for the training and test datasets, respectively. NSGA-III was then employed to derive Pareto-optimal mix designs, enabling simultaneous optimization of strength, cost, and CO_2_ emissions. To validate the proposed multi-objective optimization framework, SCBA concrete specimens were prepared using the optimized mix proportions and tested under uniaxial compression. The experimental results exhibited good agreement with the predicted values, with deviations within an acceptable margin, thereby confirming the accuracy and reliability of the framework. This study provides a practical approach for the intelligent design of low-carbon SCBA concrete, contributing to the advancement of sustainable construction practices.

## 1. Introduction

The cement industry forms the backbone of modern cities, and its total carbon dioxide equivalent (CO_2_) emissions account for 8% of the world’s emissions [1]. Each ton of cement releases 900 kg of CO_2_, and in the clinker production process, the CO_2_ directly released by calcination accounts for 50% of the cement production emissions [2]. However, with the deepening global concern over climate change and environmental impacts, the high CO_2_ emissions of the cement industry have become an urgent issue. Therefore, it is imperative to facilitate the transition of the cement industry from its conventional high-carbon emission paradigm to a low-carbon model, in alignment with the urgent global imperative to mitigate carbon emissions. To achieve this goal, a widely discussed strategy is the introduction of supplementary cementitious materials, partially replacing ordinary Portland cement, thereby reducing carbon emissions and achieving carbon neutrality in cement [3,4].

**Figure 1:**
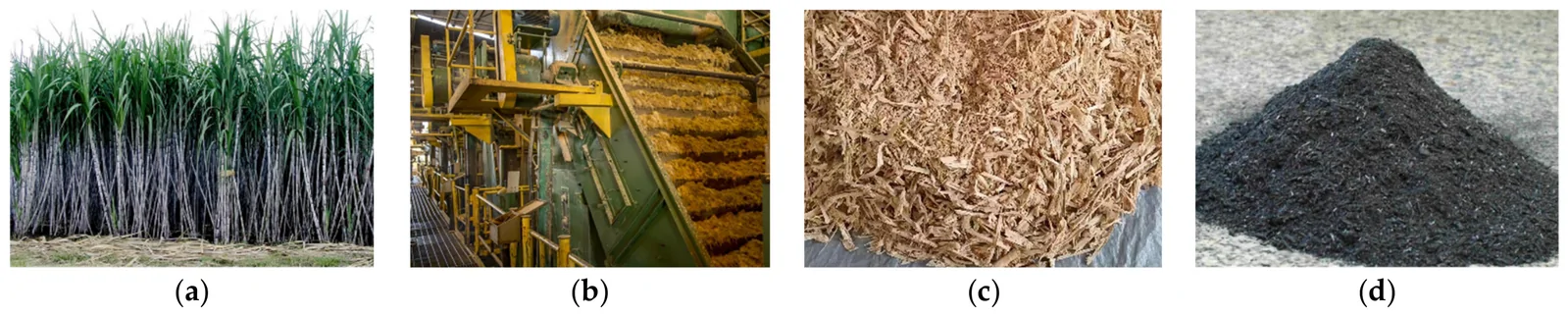
Obtaining Process of Raw SCBA. (**a**) sugarcane; (**b**) pressing; (**c**) bagasse; (**d**) raw SCBA.

Nowadays, agricultural by-products are usually used as biomass fuel for incineration into energy to meet the needs of industrial development. Biomass ash, as an end-product of the agricultural production cycle, demonstrates a consistent and increasing trend in its annual production volume. Biomass ashes such as rice husk ash [5], bamboo stem ash [6] and wheat straw ash [7], exhibiting properties akin to volcanic ash, serve as potential supplementary cementing materials for the preparation of concrete [8], bricks [9] and other building materials. Among these, sugarcane bagasse ash (SCBA) is the ash obtained by the dust removal device after the incineration of sugar byproduct bagasse. China, ranking third globally in sugarcane production, possesses a vast tropical and subtropical agricultural area. Consequently, China has a substantial and sustained supply of SCBA. The utilization of SCBA benefits from its stable and sustained supply, while the carbon dioxide emitted during SCBA combustion is reabsorbed through photosynthesis during the sugarcane growth cycle [10]. SCBA exhibits a substantial SiO_2_ content, which makes it a promising candidate for inducing pozzolanic reactivity and filling effects in concrete [11]. The SCBA concrete holds the potential for significant cost reduction, energy conservation, and optimal mitigation of waste emissions. Figure 1 depicts the process of obtaining raw SCBA.

In the application of SCBA concrete, the design of concrete mix proportions is a critical aspect due to its substantial influence on the mechanical properties and cost of concrete. At present, most concrete engineers initially establish preliminary correlations among various materials using empirical formulas when designing concrete mix proportions. Subsequently, a series of experiments were conducted to obtain suitable mix proportions [12,13]. As a supplementary cementing material, the inherent physical and chemical properties of SCBA significantly determine the compressive strength of the concrete [14,15]. There are intricate relations between these properties, leading to the inadequacy of conventional mathematical models in expressing them. Therefore, the experimental process for mix design is time-consuming and difficult to balance the strength and economy aspects of SCBA. The mix proportion design of SCBA concrete represents a typical multi-objective optimization problem. In addition, as SCBA concrete is widely recognized as a substantial material for reducing CO_2_ emissions in the concrete industry, CO_2_ emissions hold another significance in the SCBA mix proportions optimization. Researchers have explored multi-objective optimization methods focusing on CO_2_ emissions in the areas of concrete proportioning, reinforced concrete structures, and bridge construction. Liu et al. [16] evaluated the CO_2_ emissions of high-strength concrete with a high content of volcanic ash using a life cycle assessment technique and developed an index of sustainability potential for the concrete. Zhang et al. [17] adopted a multi-objective optimization algorithm to carry out sustainable design of reinforced concrete beams based on the trade-off between CO_2_ emission and cost, and the results showed that increasing the cost by 5–6% could reduce carbon emission by up to 14.7%. However, to the best of the authors’ knowledge, there has been seldom research quantifying the CO_2_ emissions of SCBA concrete, nor investigating the optimization of SCBA mix design considering CO_2_ emissions, cost factors, and mechanical properties.

Multi-objective optimization requires surrogate models that balance predictive accuracy and computational efficiency. Conventional surrogate models can generally be divided into mechanistic and numerical models. Mechanistic models have explicit formulations and high computational efficiency, but often require simplifying assumptions that limit their accuracy in representing complex structural behavior. Numerical models, such as nonlinear finite element analysis, can provide more detailed simulations; however, their substantial computational cost restricts their repeated use in complex multi-objective optimization [18]. Machine learning (ML) provides an alternative by learning nonlinear relationships directly from data and has been widely applied to predicting concrete properties [19,20], structural responses [21,22], and failure modes [23,24], etc. ML-based models are adopted as surrogate models in various multi-objective optimizations such as concrete mix design [25] and reinforced concrete flat slabs [26].

Recent studies on sustainable concrete have increasingly shifted from individual ML algorithms toward ensemble learning, hybrid models, and automated hyperparameter optimization. Previous ML studies reported a test-set of 0.9612 for a Grid Search CV–GBRT model using 164 eco-friendly concrete mixtures [27]. and a tenfold cross-validation of 0.8669 for a TPE–GBDT model using 521 recycled-aggregate concrete mixtures [28]. For agricultural-waste-based concrete, SVR achieved a test-set of 0.92 using 340 SCBA mixtures [29], while hybrid ANN models achieved 0.9709 using 192 rice-husk-ash concrete mixtures [30]. ANN and Gaussian process regression were also validated using 909 rice-husk-ash concrete samples [31]. Collectively, these findings indicate that predictive performance depends not only on the selected algorithm but also on dataset composition, sample size, validation strategy, and hyperparameter optimization. However, conventional ML still requires substantial manual effort in algorithm selection and hyperparameter tuning. Automated machine learning (Auto-ML) can automate pipeline construction, algorithm selection, and hyperparameter optimization, thereby improving modeling efficiency and reducing dependence on manual trial and error [32]. Although Auto-ML has been applied in medicine [33], agriculture [34], and architecture [35], its integration with multi-objective optimization for SCBA concrete remains limited.

**Figure 2:**
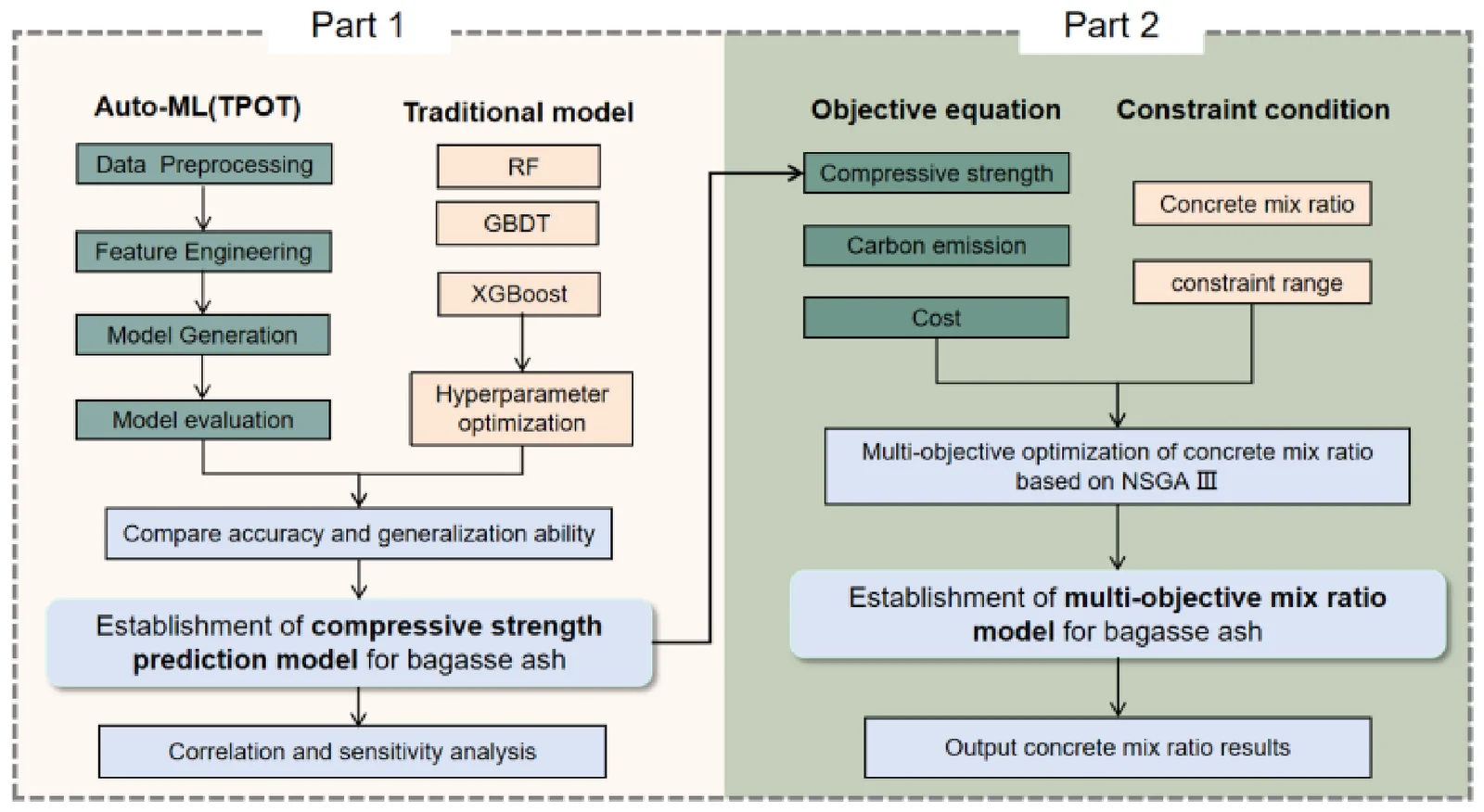
Research content and steps.

In addressing the urgent need for sustainable construction practices, this study establishes a multi-objective optimization model for SCBA concrete mix design using a combination of Auto-ML and NSGA-III. The optimization takes into account mechanical properties, CO_2_ emissions, and cost. This study mainly consists of two parts, as shown in Figure 2: (1) utilizing the Auto-ML tool TPOT to automatically generate the surrogate model for compressive strength, thereby establishing a mapping bridge between compressive strength of SCBA concrete and mix proportion parameters, and sensitivity analysis is also conducted to reveal the most influential features of the model among influential factors; (2) employing NSGA-III framework to establish a comprehensive multi-objective optimization model with compressive strength, CO_2_ emissions, and cost as multiple objectives. The final optimization results are verified and validated through uniaxial compression test results of SCBA concrete. Through these endeavors, the research contributes to the evolving discourse on sustainable construction practices, offering insights that may inform future explorations into eco-friendly building materials. It is hoped that the findings presented herein will serve as a foundation for further investigations, potentially guiding the construction industry towards more sustainable practices.

## 2. Theory and Methods

### 2.1. Machine Learning-Based Surrogate Model

#### 2.1.1. Tree-Based Pipeline Optimization Tool (TPOT) for Auto-ML

Auto-ML refers to the process of automating time-consuming and iteratively repetitive tasks in the development of ML models [36]. These tasks typically cover data preprocessing, feature engineering, model selection, hyperparameter tuning, and others. By automating these steps, there is a significant reduction in the need for manual intervention, accelerating the entire model development process while decreasing dependence on specialized ML knowledge. In practical applications, several widely used frameworks for Auto-ML simplify the handling of complex tasks. Examples include Auto-Sklearn [37], TPOT [38], Auto-Keras [39], and Auto-ml [40]. Auto-Keras focuses primarily on automating the development of neural network models and is suitable for tasks in areas such as image and text processing [41].

**Figure 3:**
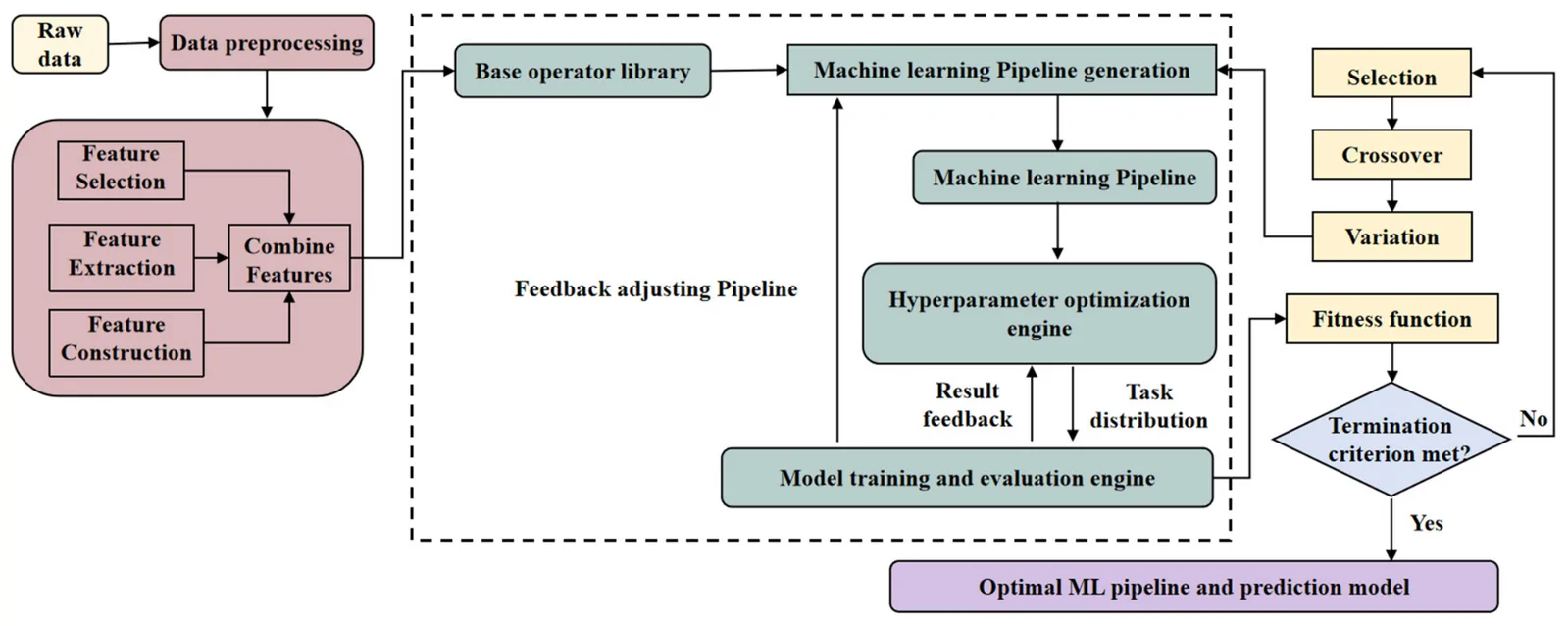
The operation steps of TPOT.

TPOT adopts a tree-based structure to represent a model pipeline for predictive modeling problems. In TPOT, genetic programming is a key optimization technique for random global search and optimization of ML model pipelines, covering data preprocessing, feature selection, transformation, and algorithm selection, as well as hyperparameter setting [42]. The operation steps of TPOT are shown in Figure 3, which are: first, it randomly creates an initial population where each individual represents a potential ML model pipeline. Secondly, it evaluates the fitness of each individual using a fitness function (such as accuracy, mean squared error, etc.), which is defined as [43]

(1) $$a=\frac{1}{n}\sum_{i=0}^{n-1}h(v_{\mathrm{predict}}=v_{\mathrm{true}})$$

where α, *n*, and *v* are the fitness function, number of samples, and value, respectively; h(x) is the indicator function. Then, based on the evaluated results, it selects excellent individuals as “parents,” applies crossover and mutation operations to generate new “offspring” individuals, gradually improving the fitness of individuals by updating the population and iterating evolution. The ML model pipelines in the population are optimized step by step. The evolution process can be terminated by the number of iterations, time limits, or performance thresholds. TPOT supports a variety of ML algorithms, including RF, SVM, KNN, GBDT, XGBoost, etc.

#### 2.1.2. Benchmark Machine Learning Models

Random Forest (RF), Gradient Boosting Decision Tree (GBDT), and Extreme Gradient Boosting (XGBoost) are selected as benchmark models for comparison with Auto-ML. RF constructs multiple decision trees using bootstrap samples and random subsets of input features and averages their predictions to reduce model variance [44,45]. GBDT sequentially fits decision trees to the residuals of preceding models, thereby progressively reducing prediction errors [46]. XGBoost extends gradient boosting by incorporating regularization and second-order gradient information to control model complexity and improve computational efficiency [47]. TPOT employs genetic programming to automatically search combinations of preprocessing procedures, regression algorithms, and hyperparameters.

In order to ensure that the selected model possesses superior accuracy, generalization capability, and reliability, it is imperative to conduct a comprehensive performance comparison between the Auto-ML model and the prediction results of RF, GBDT, and XGBoost. The objective is to identify the model that is most suitable for predicting the compressive strength of SCBA concrete.

#### 2.1.3. Evaluation Indicators

Evaluation metrics play a key role in guiding, evaluating, and improving models in ML by quantifying the difference between model predictions and actual observations. This study employs three widely utilized metrics, namely R-Square (R^2^), mean absolute error (MAE), and root mean square error (RMSE), to comprehensively evaluate the prediction performance of regression models. MAE represents the mean of the absolute difference between the predicted value and the real value, which is less affected by outliers. RMSE is employed to measure the deviation between the predicted value and the real value, which is sensitive to outliers. The closer the two indicators are to 0, the better the model fit is. R^2^ reflects the effectiveness of fitting the dependent variable to the regression relationship with the independent variable. Its values range between 0 and 1, where closer proximity to 1 indicates superior model fitting, while values closer to 0 signify poorer model performance. The MAE, RMSE, and R^2^ are given by Equations (2)–(4) [48,49].

(2) $$MAE(y,\overset{⌢}{y})=\frac{1}{m}\sum_{i=1}^{m}(\left|y_{i}-f(x_{i})\right|)$$

(3) $$RMSE(y,\overset{⌢}{y})=\sqrt{\frac{1}{m}\sum_{i=1}^{m}(y_{i}-f(x_{i}}){)}^{2}$$

(4) $$R^{2}(y,\overset{⌢}{y})=1-\frac{\sum_{i=1}^{m}{\left(y_{i}-f(x_{i})\right)}^{2}}{\sum_{i=1}^{m}{\left(y_{i}-\bar{y}\right)}^{2}}$$

where $y_{i}$ is the true value of samples, $\overset{⌢}{y}=f(x_{i})$ is the predicted values of the model, $\bar{y}$ is the average of the true values of samples, and m is the number of samples.

#### 2.1.4. Hyperparameter Optimization

Hyperparameters control model complexity and learning behavior, and are specified before model training. For RF, n_estimators specifies the total number of trees, max_depth limits the maximum depth of each tree, min_samples_split defines the minimum number of samples required to split an internal node, and min_samples_leaf defines the minimum number of samples required at a leaf node. GBDT uses these four hyperparameters together with learning_rate, which controls the contribution of each boosting tree. XGBoost uses n_estimators, max_depth, and learning_rate, together with min_child_weight, which specifies the minimum sum of instance weights required in a child node, and gamma, which specifies the minimum loss reduction required for an additional split.

Common hyperparameter optimization methods include grid search [50], random search [51], and Bayesian optimization [52]. In this study, grid search combined with five-fold cross-validation [53] is employed to optimize RF, GBDT, and XGBoost. The training set is divided into five approximately equal folds. In each iteration, four folds are used for model fitting, while the remaining fold is used for validation. The mean MAE across the five validation folds is used for selecting the optimal hyperparameter configuration, while the independent testing set is not involved in hyperparameter selection. For Auto-ML, TPOT employs genetic programming to automatically search candidate regression algorithms and their hyperparameters using the cross-validated MAE as the fitness criterion.

(5) $$CV_{\left(5\right)}=\frac{1}{5}\sum_{i=1}^{5}MAE_{i}$$

where i is the number of the data set, is the average value of multi-fold cross-validation, and $MAE_{i}$ is the MAE value of the prediction result of the i-th data set.

### 2.2. Multi-Objective Optimization Method

#### 2.2.1. Non-Dominated Sorting Genetic Algorithm III (NSGA-III)

**Figure 4:**
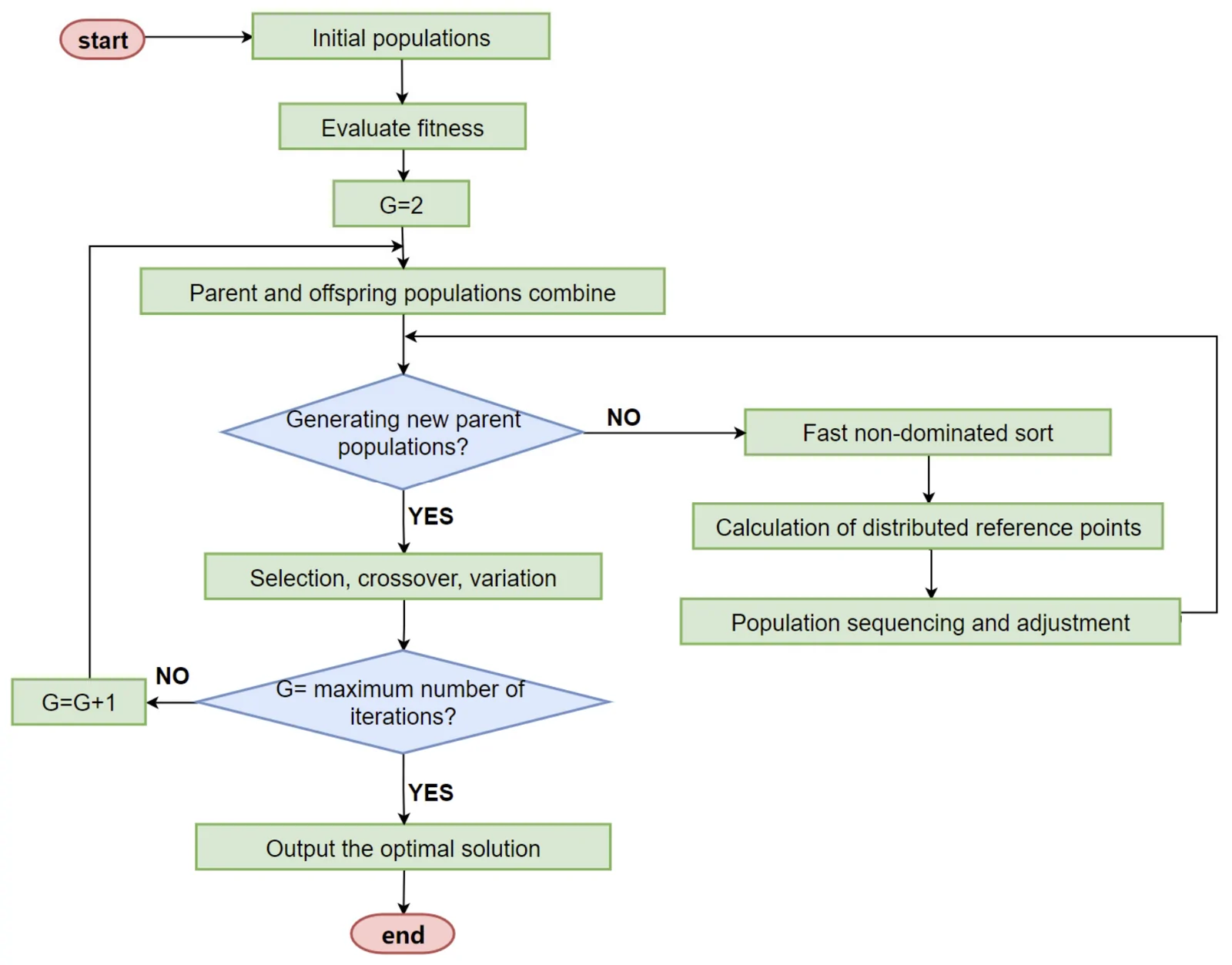
The steps of the NSGA-III algorithm.

Due to potential inherent conflicts among different objectives, optimizing one objective often comes at the expense of degrading others. Consequently, finding a unique optimal solution for multi-objective optimization problems is difficult. To overcome this challenge, it is necessary to identify solutions that cannot be simultaneously outperformed or are at least as good as other solutions on all objectives. These solutions form the Pareto front, which represents the set of optimal trade-off solutions in multi-objective optimization problems. NSGA-II and NSGA-III are both multi-objective optimization algorithms based on Pareto optimal solutions, aiming to discover diverse and evenly distributed Pareto front sets. The difference between NSGA-II and NSGA-III lies in their selection mechanisms. NSGA-II employs a crowding distance to select individuals within the same non-dominated rank, while NSGA-III uses a reference-point-based method to select individuals. When dealing with multi-objective optimization problems involving three or more objectives, the crowding distance method can lead to a decrease in the convergence and diversity of the algorithm, causing it to get trapped in local optima. NSGA-III is applicable to various application fields, including engineering design [54], resource allocation [55], path planning [56], and optimization. The steps of the NSGA-III algorithm are presented in Figure 4, which are summarized as follows [57]:

(1) Randomly generate a population of size N as the initial parent generation, setting the current iteration number. For each individual in the population, calculate its fitness values on each objective function. The offspring individuals generated through selection, crossover, and mutation operations combine to form a new offspring population.
(2) Combine the offspring and parent populations to obtain a population of size 2N. Traverse the population, compare each individual, and determine its non-dominated relationships and non-dominated ranks. Subsequently, sort the partitioned individuals in descending order based on non-dominated ranks and store them in a new population until the size of the new population reaches N or exceeds it for the first time. Typically, the solutions in the last non-dominated rank are only partially accepted.
(3) NSGA-III uses a reference point-based approach to truncate solutions in the last non-dominated rank. The selection process includes the determination of the reference point P on the hyperplane, the standardization of the objective function, and the calculation of the minimum distance between the population individuals and the reference point. According to the number of reference points being selected, the one with fewer reference points is chosen as the basis for the selection of individuals in the last non-dominated rank of the population, thus realizing the acquisition of the solution [57].

(6) $$P=C_{M+H-1}^{H}$$

(7) $$f_{i}^{n}(x)=f_{i}^{\prime }(x)/(a_{i}-z_{i}^{\min})=(f_{i}^{\prime }(x)-z_{i}^{\min})/(a_{i}-z_{i}^{\min}),i=1,2,\ldots ,M$$

(8) d(S)=d⊥(S,π(S))

where M is the dimension of the target space, representing the number of optimization objectives. Each object is divided into H parts and the number of reference points is P, ${f^{\prime }}_{i}(x)$ is the i-th transformation object, a is the intercept of the hyperplane with the coordinate axes; $\bar{z}=(z_{1}^{\min},z_{2}^{\min},\ldots ,z_{M}^{\min})$ is the constructed ideal point, $f_{i}^{n}$ is a transformation formula for the objective function, S is the hyperplane, π(S) is the reference point, and d is the minimum distance between individuals in the population and the reference points.

(4) The new population is taken as the parent population, and selection, crossover, mutation, and other operations are performed to obtain a new population of offspring.
(5) Repeat steps (2)–(4) until the maximum number of iterations is reached, or the target is sufficiently close to the ideal solution, and finally, the Pareto optimal solution is obtained.

#### 2.2.2. Formulation of Objective Functions

The objective functions in NSGA-III serve to quantify objectives. By leveraging the values of the objective functions, NSGA-III can effectively perform non-dominated sorting, selection, crossover, mutation, and other operations to search for the Pareto front (non-dominated solution set) of multi-objective optimization problems, thereby providing a diverse and balanced set of solutions. The objective functions applied in this study are given as follows:

(1) Compressive strength of SCBA concrete.

Auto-ML establishes a mapping relationship between input variables and output variables based on an existing dataset. The compressive strength prediction model output by Auto-ML is transformed into the objective function for the compressive strength of SCBA concrete.

(9) $$F1=h(x_{1},x_{2},\ldots ,x_{n})$$

where $x_{1},x_{2},\ldots ,x_{n}$ represent the input variables of Auto-ML, and $h(x_{1},x_{2},\ldots ,x_{n})$ is the expression for the mapping relationship between the ML features and the output values.

(2) CO_2_ emissions of SCBA concrete

**Figure 5:**
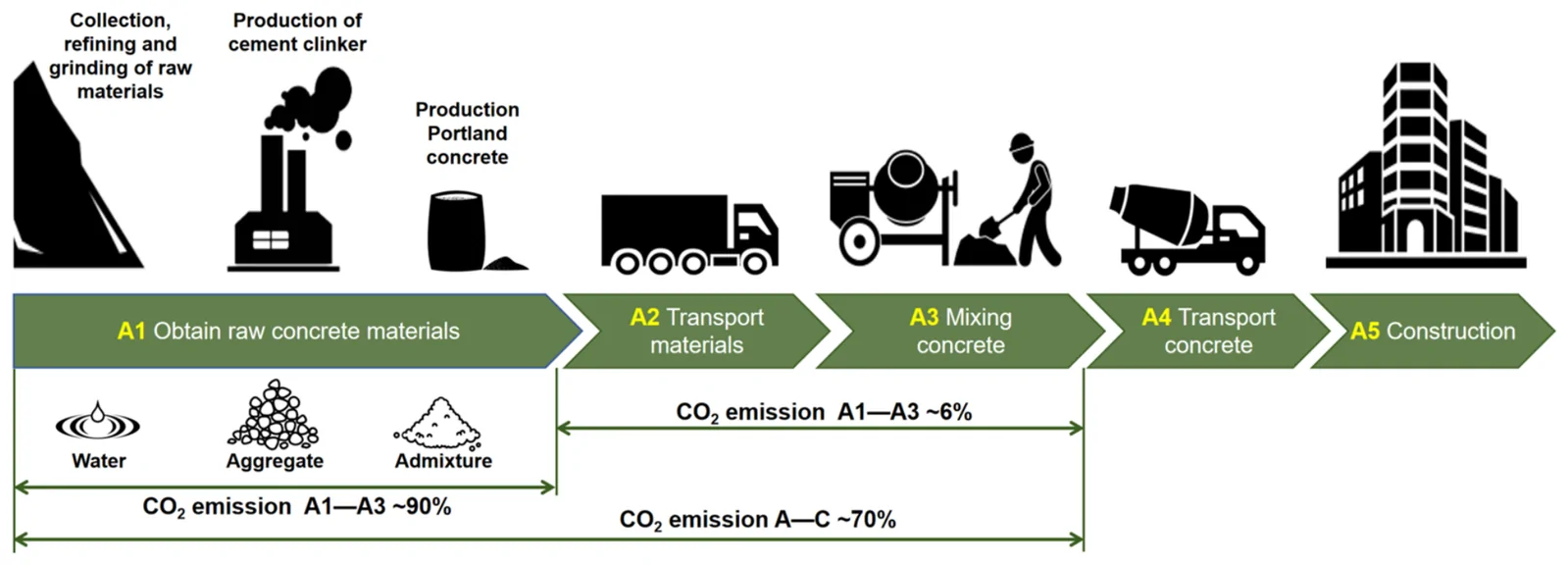
The main sources of CO_2_ emissions in stage A.

Concrete’s CO_2_ emissions throughout its full life cycle can be roughly divided into four stages: A-D. Among them, A represents the “pre-production stage”; B is the “use stage”; C is the “end-of-life stage”; and D is the “post-lifecycle stage” [58]. Figure 5 illustrates the main sources of CO_2_ emissions in stage A, where the CO_2_ emissions generated in stages A1-A3 account for about 75% of the CO_2_ emissions in stages A-C of concrete’s full life cycle [59]. Globally, the raw materials used for concrete production are abundant and locally supplied. Therefore, the proportion of CO_2_ emissions during the raw materials and concrete transportation stages is low. This study does not consider the CO_2_ emissions generated during the transportation stage.

The total CO_2_ emissions are calculated based on the combined contributions of the carbon emissions ${CO}_{2}\left(M\right)$ related to the acquisition and manufacturing stages of the raw materials for SCBA concrete per cubic meter and the carbon emissions during the concrete mixing stage. This calculation also takes into account the grinding and sieving of materials. The objective function F2 is formulated to compute the total CO_2_ emissions.

(10) $${CO}_{2}\left(M\right)=\sum_{i=1}^{n}m_{i}E_{i}$$

(11) $${CO}_{2}\left(P\right)=TQ$$

(12) $$F2={CO}_{2}\left(M\right)+{CO}_{2}\left(P\right)$$

where $m_{i}$ represents the consumption of raw materials per cubic meter of concrete preparation, $E_{i}$ is the carbon emission factor of the raw materials, *T* denotes the total electricity consumption associated with grinding, sieving, blending the cementitious materials, and mixing the SCBA concrete, and *Q* is the carbon emission factor of electricity. *M* and *P* represent the raw-material and production stages, respectively.

(3) Cost of SCBA concrete

Material costs significantly influence the economic performance of concrete. The economic cost per cubic meter of SCBA concrete is obtained by establishing a polynomial based on the unit price and consumption of raw materials. The objective function F3 for the cost of SCBA concrete is formulated as follows:

(13) $$F3=\sum_{i=1}^{n}m_{i}\times UP_{i}$$

where $m_{i}$ represents the quantity of materials consumed in preparing one cubic meter of concrete, and $UP_{i}$ is the unit price of the raw materials.

#### 2.2.3. Pareto Frontier Solutions

**Figure 6:**
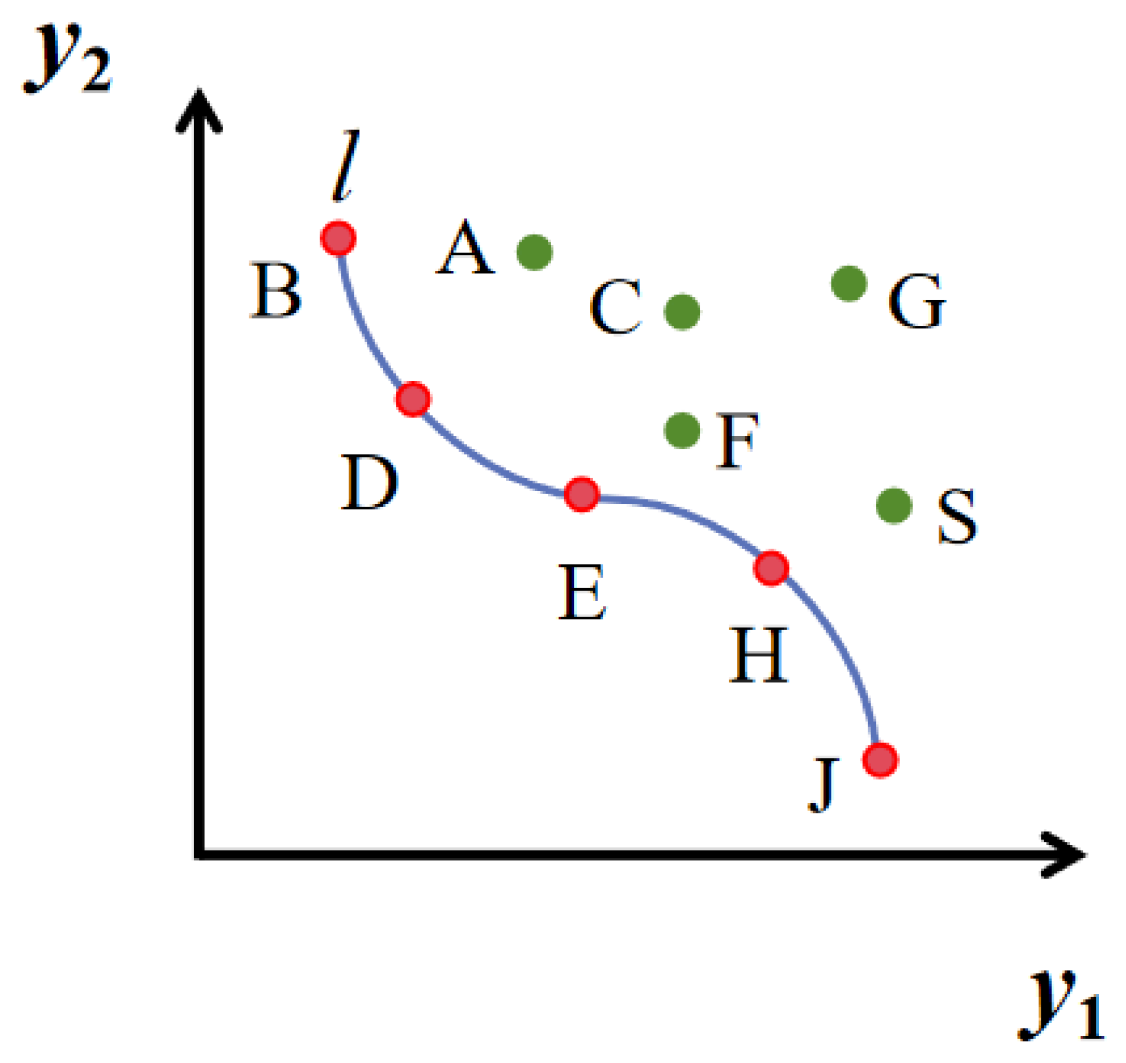
Pareto optimal frontier. Here, *y*_1_ and *y*_2_ are minimized objectives; A–J denote candidate solutions, with red and green indicating non-dominated and dominated solutions, respectively.

Pareto optimality is a state that describes how resources are best allocated. In this state, no party can gain more benefits without harming any other party involved in the allocation of resources. There may be many Pareto optimal solutions, and they constitute a range within which it is impossible to make one goal better without harming the others, no matter how we adjust our decisions. The Pareto frontier is a surface formed in space by the set of optimal solutions of the objective function. If it is an optimization problem with two objectives, the Pareto frontier is usually a line, as shown in Figure 6, where B, D, E, H, and J are the optimal solutions of the objective function, and line l is the Pareto frontier. In the case of multiple targets, the Pareto front is usually a curved surface.

## 3. Case Study

In order to validate the proposed Auto-ML surrogate model and multi-objective optimization model, this paper introduces a case study involving the multi-objective optimization of SCBA concrete. The optimized mix proportions are validated by compressive tests of SCBA concrete.

### 3.1. Prediction of Compressive Strength of SCBA Concrete

#### 3.1.1. Dataset of SCBA Concrete

The dataset utilized in this study comprises 162 sets of data from previously published literature [11,60,61,62,63,64,65,66,67,68,69,70,71,72,73,74] (for detailed information, refer to Appendix A). The ML features comprise the mix design parameters of SCBA concrete, including the relative mass replacement rate of SCBA (*R*_SCBA_/%), cement content (*C*/kg/m^3^), water–cement ratio (*w*/*c*), fine aggregate (*FA*/kg/m^3^), and coarse aggregate (*CA*/kg/m^3^). The output parameter is the compressive strength of SCBA concrete at 28 days (*f*_c_/MPa). Statistics for the dataset regarding input and output variables are presented in Table 1.

The data samples are divided into training and testing sets in a ratio of 0.8:0.2 [75]. The statistical comparability of the training and testing sets is assessed by comparing the descriptive statistics of all input variables and compressive strength. In addition, the two-sample Kolmogorov–Smirnov test (KS) [76], Levene’s test [77], and standardized mean difference (SMD) [78] are used to evaluate differences in distribution, variance, and mean, respectively. As shown in Table 2, all KS and Levene’s tests yield *p*-values above 0.05, while the absolute SMD values range from 0.0007 to 0.1537, below the small-effect reference value of 0.20 proposed by Cohen [79]. These results indicate that no substantial imbalance is detected between the training and testing sets.

In general, the varying scales and units of different features can impact the results of data analysis. To address this issue, this paper employs a data normalization method. The preprocessed data is constrained within a specific range [0,1] using Equation (1), aiming to eliminate adverse effects caused by differences in scale between features and outliers [80].

(14) $$x^{\prime }=\frac{x-\min(x)}{\max(x)-\min(x)}$$

where $x^{\prime }$ denotes the data after feature value normalization, max(x) represents the maximum value of the sample data, and min(*x*) represents the minimum value of the sample data.

#### 3.1.2. Determination of Hyperparameters

Defining the range of hyperparameter values for ML models, a combination of grid search and five-fold cross-validation is employed for hyperparameter tuning. Optimization of hyperparameters is based on the average error of test set predictions from five-fold cross-validation, serving as the final evaluation metric. Table 3 illustrates the hyperparameter values for RF, GBDT, XGBoost, and TPOT. The hyperparameters of the AUTO-ML model are automatically selected, which are also given in Table 3.

#### 3.1.3. Analysis of Prediction Results

**Figure 7:**
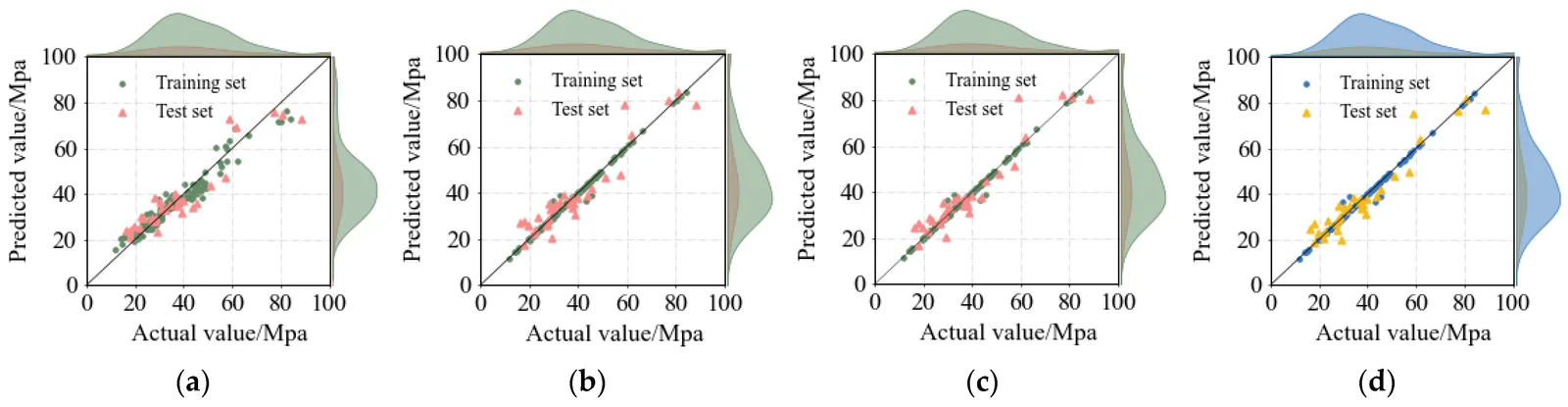
The prediction results of the four machine learning models. (**a**) RF; (**b**) GBDT; (**c**) XGBoost; (**d**) Auto-ML.

The optimized RF, GBDT, XGBoost, and the Auto-ML models are employed to predict the compressive strength of SCBA concrete. Figure 7 illustrates the prediction results of the four ML models, presenting scatter plots and density curve plots. The *x*-axis represents the actual observed values, while the *y*-axis represents the model’s predicted values, with each data point representing a sample. The marginal density plots at the upper and right edges show the distributions of the actual and predicted values, respectively, for the training and test sets. It is important to note that there is a baseline in the graph, representing the scenario where the predicted results are exactly equal to the actual results. The closer the points are to the baseline, the better the predictive performance of the model. Observing the density curve plots in Figure 7, we can see that the training set data points are widely distributed in the graph, mainly within the range of 15–65 MPa, while the testing set is concentrated in the range of 20–60 MPa. Most data points in the graph are clustered around the baseline, indicating relatively good predictive performance for all four models. Data points in Figure 7b are relatively more scattered compared to the other three plots, suggesting that the GBDT model has slightly inferior predictive performance. On the other hand, data points in Figure 7d are closer to the baseline, indicating that the Auto-ML model possesses high accuracy and excellent generalization performance.

Table 4 presents the performance metrics for four ML models in terms of predictive performance. All models achieve training values above 0.9, indicating strong fitting capabilities. However, their performance declines on the testing set, with gaps of 0.064, 0.108, 0.100, and 0.085 for RF, GBDT, XGBoost, and Auto-ML, respectively. The substantially lower training errors than testing errors, particularly for GBDT, XGBoost, and Auto-ML, suggest potential overfitting. Nevertheless, Auto-ML achieves the highest testing accuracy (0.908) and the lowest testing MAE (4.057) and RMSE (5.446), demonstrating the best predictive performance on the held-out testing set. This performance is comparable to previous studies: an SVR model developed using 340 SCBA concrete mixtures achieved an R^2^ of 0.92 [29], while a hybrid ANN based on 192 rice-husk-ash concrete mixtures achieved an R^2^ of 0.97 [30]. Therefore, Auto-ML is selected as the surrogate model, and the optimization variables are restricted to the ranges of the original dataset to avoid extrapolation.

#### 3.1.4. Correlation and Sensitivity Analysis

**Figure 8:**
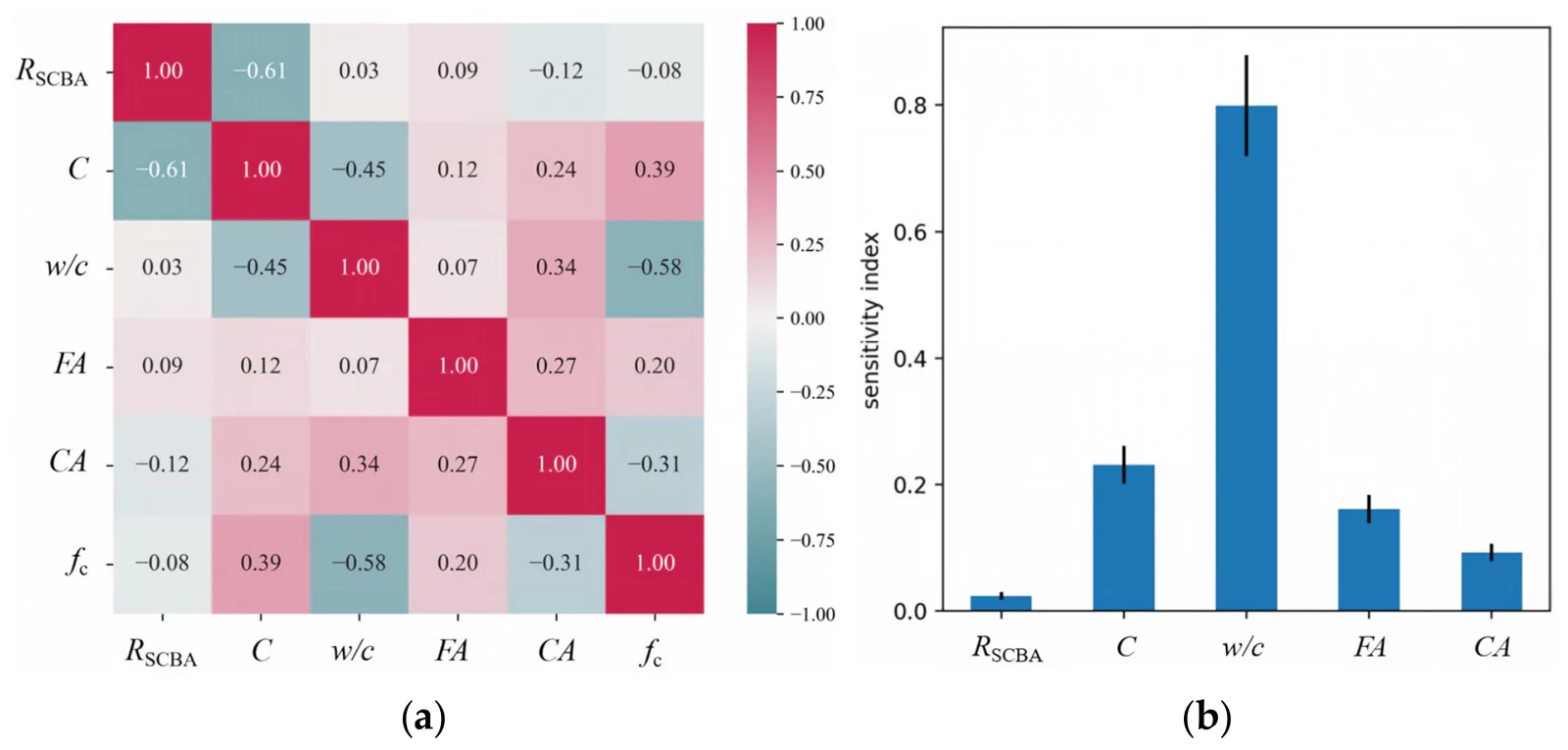
The relationship between output variables and input variables. (**a**) Pearson correlation analysis; (**b**) sensitivity analysis based on Auto-ML.

Pearson correlation analysis and sensitivity analysis based on Auto-ML are conducted on the collected data to assess the impact of five SCBA concrete mix design parameters on compressive strength. The sensitivity of each input parameter is expressed using sensitivity indices. The Pearson correlation analysis in Figure 8a indicates that cement content (*C*), water–cement ratio (*w*/*c*), and coarse aggregate content (*f*_c_) have significant influences on the compressive strength of SCBA concrete. Specifically, cement content shows a positive correlation with compressive strength, while the water–cement ratio and coarse aggregate content exhibit negative correlations.

The sensitivity analysis in Figure 8b identifies the water-to-cement ratio (*w*/*c*) as the most influential parameter affecting the compressive strength of SCBA concrete. This finding is consistent with Alhakeem et al. [27], who identified the water-to-binder ratio as one of the most influential factors affecting concrete strength. For SCBA concrete, Javed et al. [81] reported that cement content was the most influential variable, followed by *w*/*c*. Differences in the relative rankings may result from variations in mixture ranges, material properties, and dataset composition; nevertheless, both studies confirm the importance of the binder–water balance in strength development.

### 3.2. Multi-Objective Optimization of SCBA Concrete

#### 3.2.1. Establishment of Objective Functions

(1) The objective function for the compressive strength of SCBA concrete.

The predictive model for the compressive strength of SCBA concrete has been established in Section 3.1 and is denoted by Equation (9), representing the objective function F1 for the compressive strength of SCBA concrete.

(15) $$F1=\max[h\left(x_{1},x_{2},x_{3},x_{4},x_{5}\right)]$$

where $x_{1},x_{2},x_{3},x_{4},x_{5}$ are the SCBA proportion, cement content, water–binder ratio, fine aggregate content, and coarse aggregate content in Table 1, respectively.

(2) The objective function for carbon emissions of SCBA concrete.

Calculation of the objective function F2 for total carbon dioxide emissions per cubic meter of SCBA concrete according to Equation (16).

(16) $$F2=\min(m_{1}E_{1}+m_{2}E_{2}+m_{3}E_{3}+m_{4}E_{4}+m_{5}E_{5}+TQ)$$

where $m_{i}$ represents the consumption of five raw materials per cubic meter of concrete, $E_{i}$ denotes the carbon emission factors for cement (0.83 ${\mathrm{kgCO}}_{2}/\mathrm{kg}$), water (0.0003 ${\mathrm{kgCO}}_{2}/\mathrm{kg}$), fine aggregate (0.0050 ${\mathrm{kgCO}}_{2}/\mathrm{kg}$), and coarse aggregate (0.0062 ${\mathrm{kgCO}}_{2}/\mathrm{kg}$), determined based on previous research [82]. T and Q represent the electricity consumption and the carbon emission per unit of electricity for grinding, sieving cementitious materials, and mixing SCBA concrete, set at 3.82 $(\mathrm{kw}\cdot {h)/m}^{3}$ and 0.77 CO_2_ $(\mathrm{kw}\cdot {h)/m}^{3}$ [83].

(3) The objective function for the economic cost of SCBA concrete.

Establishing a polynomial based on the unit price and consumption of the five raw materials to obtain the economic cost of SCBA concrete per cubic meter. Substituting into Equation (17) yields the objective function F3 for the cost of SCBA concrete:

(17) $$F3=\min\left(m_{1}\times UP_{1}+m_{2}\times UP_{2}+m_{3}\times UP_{3}+m_{4}\times UP_{4}+m_{5}\times UP_{5}\right)$$

where $m_{i}$ represents the consumption of five raw materials for preparing one cubic meter of concrete; $UP_{i}$ denotes the unit price of SCBA, cement, water, fine aggregate, and coarse aggregate, which is chosen as 0.09, 0.35, 0.002, 0.125, and 0.102 RMB/kg, respectively [84].

#### 3.2.2. Constraint Condition

According to the requirements of the code for design of concrete structures [85] and engineering practices, the dosage of each component in the mix proportion of SCBA concrete should fall within a reasonable range, and the concrete strength should meet certain specifications in Table 5.

Where *R*_SCBA_, C, *w*/*c*, FA, CA are SCBA proportion, cement content, water–binder ratio, fine aggregate content and coarse aggregate content, respectively; $f_{cu,0}$ is the preparation strength of concrete during construction, $f_{cu,k}$ is the standard value of compressive strength of designed concrete cubes, σ is the standard deviation of concrete strength during construction, 5 MPa for C30 and C40, and 6 MPa for C50.

#### 3.2.3. Results and Analysis

NSGA-III is a multi-objective optimization method based on genetic algorithms. Common parameters for NSGA-III include population size, maximum number of iterations, mutation probability, crossover probability, and offspring population size. The selection of population size depends on the complexity of the specific problem; the maximum number of iterations controls the runtime of the algorithm; mutation probability helps maintain the diversity of the population, aiding the algorithm in escaping local optima. After consulting relevant literature for the initial setup [86,87] and fine-tuning through experimentation, the parameters are adjusted to optimize the model, as shown in Table 6. A multi-objective optimization model combining Auto-ML with NSGA-III is established to obtain 33 sets of SCBA concrete mix proportions, along with the corresponding compressive strength, carbon emissions, and cost.

**Figure 9:**
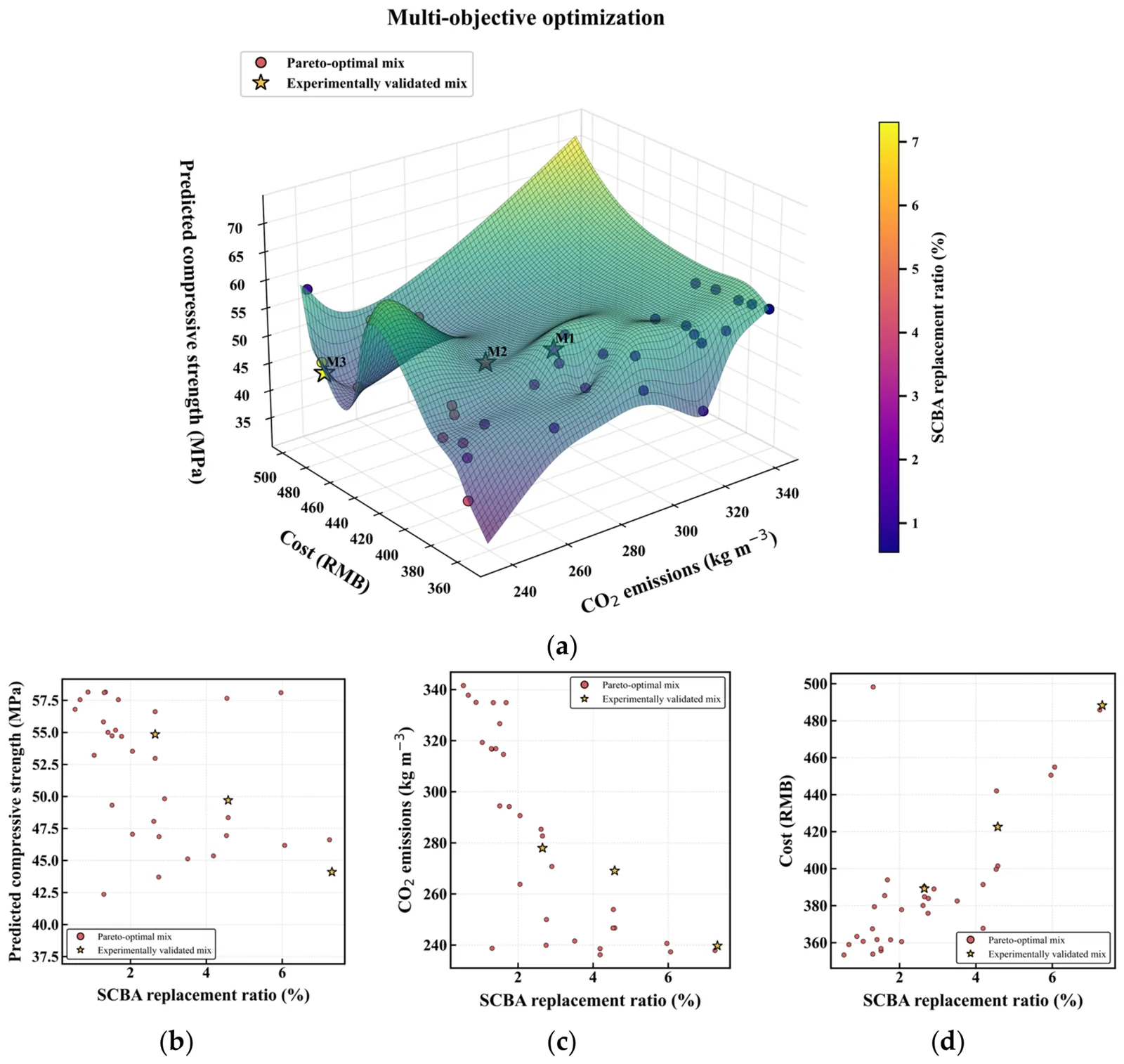
Multi-objective optimization results for SCBA concrete. (**a**) 3D view of the Pareto solution set for three-objective optimization; relationships between SCBA replacement ratio and (**b**) strength, (**c**) CO_2_ emissions, and (**d**) cost.

Figure 9 presents a three-dimensional view of the Pareto solution set composed of the 33 solution sets, with the axes representing carbon emissions, cost, and compressive strength. The solutions exhibit predicted strengths of 37.93–58.15 MPa, CO_2_ emissions of 236.22–341.54 kg/m^3^, and costs of 353.35–498.29 RMB/m^3^. Each point represents a non-dominated mixture, with its color indicating the SCBA replacement ratio, which ranges from 0.54% to 7.31%. The surface color represents the interpolated compressive strength, changing from dark blue to green as the strength increases. The Pareto-optimal solutions reveal that similar strength levels can be achieved through different combinations of cost and CO_2_ emissions. This is consistent with previous machine-learning-based concrete mixture optimization, which also produced Pareto fronts representing alternative compromises among competing objectives [88]. Some high-strength mixtures exhibit relatively low CO_2_ emissions but higher costs, whereas others exhibit higher CO_2_ emissions but lower costs. Therefore, increasing compressive strength does not necessarily reduce both economic and environmental performance simultaneously; instead, the solutions demonstrate different trade-offs among strength, cost, and CO_2_ emissions. Figure 9b–d show that the predicted strength decreases non-monotonically with the SCBA replacement ratio, reflecting the combined influence of the other mixture variables. In contrast, increasing SCBA replacement is generally associated with lower CO_2_ emissions but higher costs, revealing a clear environmental–economic trade-off among the Pareto-optimal mixtures.

### 3.3. Test Verification

Every point on the Pareto optimal front obtained through multi-objective optimization is a non-dominated solution based on the defined objectives. In this scenario, it is impossible to improve one objective without compromising others, as mix proportions with high compressive strength also entail high costs. Each point found through multi-objective optimization represents an optimal trade-off among multiple objectives. To validate the accuracy of the Auto-ML–NSGA-III multi-objective optimization model for SCBA concrete mix design, three Pareto-optimal mixtures were randomly selected from the solutions shown in Figure 9 for compressive strength testing, as highlighted by the star-shaped markers. Their mix proportions are listed in Table 7. When the replacement ratio of SCBA increases, the cement consumption decreases, leading to a reduction in carbon emissions, albeit with a decrease in the compressive strength of concrete. The additional processing of SCBA results in an increase in production costs. Consequently, with the increase in the replacement ratio, the overall cost of concrete also correspondingly increases.

#### 3.3.1. Experimental Material

**Figure 10:**
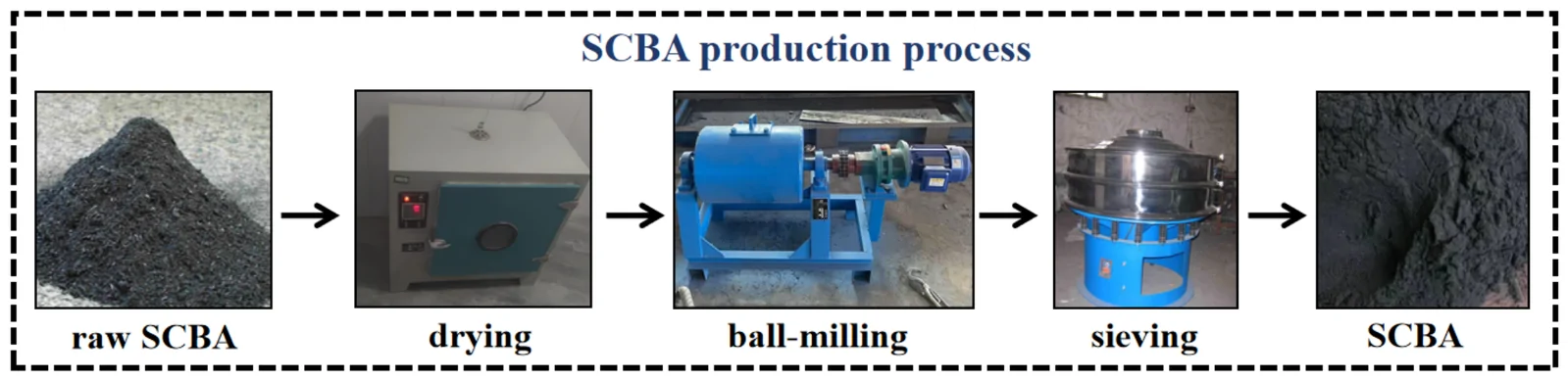
The production process of SCBA.

The proposed multi-objective optimization model based on Auto-ML is applied to the mix design of SCBA concrete. Figure 10 illustrates the production steps of SCBA and the compressive strength testing process for SCBA concrete. The raw SCBA is obtained from a sugar factory located in Jiangsu Province, China, and is a biomass ash produced through the combustion of sugarcane bagasse after the pressing process. However, upon acquisition from the factory, the SCBA exhibits a wide particle size distribution and a certain level of moisture content. Consequently, in the laboratory, the raw SCBA undergoes drying and a 2-h ball-milling process. Ultimately, the SCBA with particle sizes smaller than 75 μm for experimentation is obtained through filtration using a 200-mesh sieve [89]. The ground SCBA has a specific surface area of 0.98 m^2^/g and a specific gravity of 2.30. In concrete production, the cementitious material chosen is ordinary Portland cement with a strength class of PO42.5. Natural river sand with a maximum particle size of 2 mm is used as the fine aggregate, while crushed stone with a particle-size range of 5–20 mm is used as the coarse aggregate.

#### 3.3.2. Experimental Process and Results

**Figure 11:**
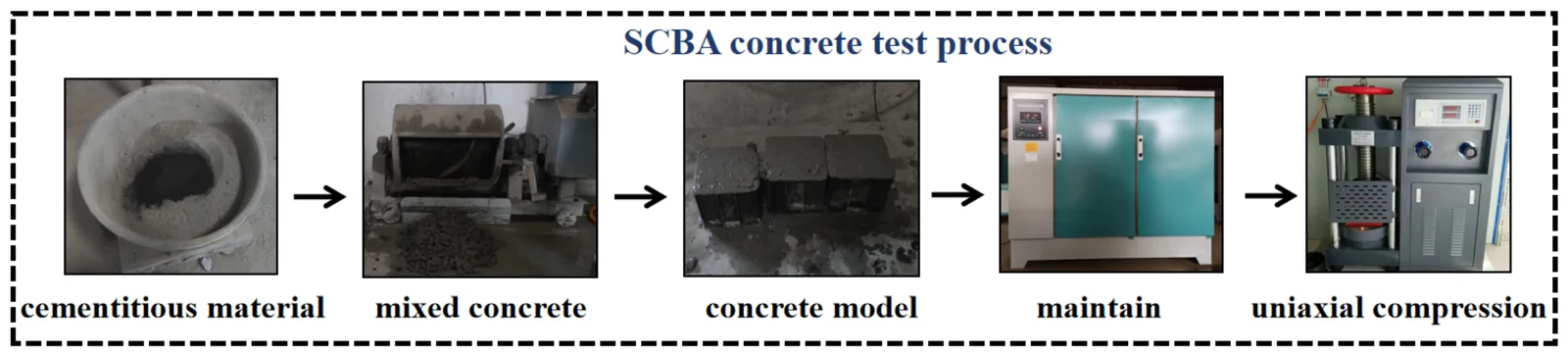
The experimental process of SCBA concrete.

In accordance with the three sets of SCBA concrete mix proportions listed in Table 6, compressive strength tests are conducted. Specimen preparation, curing, and compressive-strength testing are conducted in accordance with GB/T 50081-2019 [90]. The constituent materials are mixed in a concrete mixer, and the resulting concrete mixture is subsequently tested for slump and cast into 150 × 150 × 150 mm cube molds. The experimental procedure for the preparation and testing of SCBA concrete is shown in Figure 11. The specimens are placed on a vibrating table and vibrated until the concrete surface oozes slurry, at which point the vibration is stopped. Excess concrete is then removed, and the surface is leveled using a trowel. The specimens are then placed in a curing chamber with a relative humidity of 95% and a temperature maintained at 20 ± 1 °C for 24 h before demolding. Subsequently, the specimens are further cured under the same conditions for an additional 27 days. The compressive strength is evaluated using a TYE-2000B compression-testing machine. The average compressive strength of the three specimens is recorded for each mix proportion, representing the compressive strength of SCBA concrete under different ratios. Table 8 shows the compressive strength test results of SCBA concrete.

Table 8 displays the compressive strength of three sets of SCBA concrete specimens with different mix proportions, namely 47.72 MPa, 44.76 MPa, and 38.54 MPa. The experimental compressive strengths of the three sets of mix proportions SCBA concrete are observed to be lower than the predicted values, with discrepancies between the experimental data and corresponding predicted values for these three groups of SCBA concrete of 14.96%, 11.06%, and 14.43%, respectively. It can be concluded that, due to the inherent variability and randomness in concrete, the experimental results exhibit a small discrepancy when compared to the predicted value, which indicates that the proposed multi-objective optimization model possesses an acceptable accuracy. Consequently, this model demonstrates the potential to proficiently handle the tasks of mix proportion design and optimization for SCBA concrete.

## 4. Conclusions

Through the recycling and reuse of SCBA, partially substituting traditional cement in concrete production, there are potential advantages such as enhancing resource utilization efficiency, reducing carbon dioxide emissions, and alleviating the environmental burden of waste. However, the application environments of SCBA concrete vary, necessitating the intelligent development of its mix design methods. In this study, with the objectives of improving compressive strength, minimizing carbon emissions, and reducing costs, we construct a multi-objective optimization framework for SCBA concrete mix design based on the combination of Auto-ML and NSGA-III. The framework comprises the following components: (1) employing Auto-ML to establish a mapping function between compressive strength and mix design parameters for SCBA concrete; (2) considering the influence of complex factors in the mix design of SCBA concrete, utilizing the NSGA-III algorithm to establish a comprehensive optimization model with compressive strength, carbon emissions, and costs as multiple objectives.

Through the case analysis of 163 groups of SCBA concrete experimental test data, the following conclusions are obtained:

(1) Auto-ML is applied to establish a predictive model for the compressive strength of SCBA concrete, which serves as a surrogate model for compressive strength in multi-objective optimization. The Auto-ML model demonstrates excellent performance in predicting the compressive strength of SCBA concrete, with an R2 value of 0.908 on the test set, which is higher than the R2 values of RF, GBDT, and XGBoost. Both the MAE and RMSE values of the Auto-ML model on the training and test sets are smaller than those of the other three ML models, indicating that the prediction results of the Auto-ML model have a smaller margin of error compared to the experimental results.
(2) NSGA-III is applied for the multi-objective optimization of the mix proportion, which successfully identifies the Pareto-optimal set for SCBA concrete. This set encompasses considerations of compressive strength, cost, and CO_2_ emissions. In this study, a total of 33 Pareto solution sets are obtained, among which 3 sets are selected for experimental validation.
(3) Three sets of optimized mix proportions are selected to prepare SCBA concrete. Uniaxial compressive tests are conducted on the SCBA concrete samples. It is observed that the experimental compressive strengths of the 3 sets of mix proportions SCBA concrete are lower than the predicted values. However, since the errors between the experimental results and predicted values are all below 15%, taking into account the inherent variability and randomness in concrete, it can be concluded that the proposed multi-objective optimization model possesses an acceptable level of accuracy. It is also shown that the proposed model has the potential to effectively address the challenges related to mix proportion design and optimization for SCBA concrete.

## Figures and Tables

**Table 1:** Statistics on a dataset of input and output variables.

Mix Design	*R*_SCBA_/%	*C*/kg/m^3^	*w*/*c*	*FA*/kg/m^3^	*CA*/kg/m^3^	*f*_c_/MPa
Maximum value	50	560	0.6	1010	1288	88.35
Minimum value	0	116	0.3	240	490	15.30
Average value	14.475	327.199	0.476	673.985	1063.371	37.913
Standard deviation	11.681	82.890	0.065	159.403	176.264	14.223
Coefficient of variation pct	79.194	25.340	13.850	23.737	16.662	37.514
Q1_25pct	5.000	277.800	0.424	611.892	945.000	28.278
Median	15.000	330.000	0.500	719.000	1106.500	34.956
Q3_75pct	20.000	378.075	0.505	745.000	1174.024	43.867
Skewness	0.666	−0.132	−0.578	−0.941	−1.541	1.497
Excess kurtosis	0.080	0.185	−0.197	1.488	3.048	2.541

**Table 2:** Statistical comparison and representativeness assessment of the training and testing datasets.

Variable	*R*_SCBA_/%	*C*/kg/m^3^	*w*/*c*	*FA*/kg/m^3^	*CA*/kg/m^3^	*f*_c_/MPa
Train mean	14.777	327.690	0.472	675.222	1063.267	37.475
Train SD	12.291	82.361	0.063	153.704	169.858	13.028
Test mean	14.643	324.805	0.473	656.827	1036.347	39.665
Test SD	9.019	86.476	0.075	182.772	201.826	18.430
SMD	0.011	0.035	−0.001	0.115	0.152	−0.154
Absolute SMD	0.011	0.035	0.001	0.115	0.152	0.154
KS	0.650	0.445	0.445	0.579	0.722	0.790
Levene	0.053	0.846	0.200	0.336	0.370	0.167

**Table 3:** Model hyperparameter values.

Model	Hyperparameter	The Average of MAE
RF	max_depth = 15, min_samples_leaf = 1, min_samples_split = 2, n_estimators = 100	2.370
GBDT	learning_rate = 0.2, max_depth = 7, min_samples_leaf = 1, min_samples_split = 10, n_estimators = 80	0.484
XGBoost	learning_rate = 0.1, max_depth = 5, min_child_weight = 1, n_estimators = 100	0.437
Auto-ML	XGBoost (gamma = 0.1, learning_rate = 0.2, max_depth = 6, min_child_weight = 1, n_estimators = 100)	0.361

**Table 4:** The performance metrics for four distinct machine learning models.

Model Data	RF		GBDT		XGBoost		Auto-ML	
	Training Set	Test Set	Training Set	Test Set	Training Set	Test Set	Training Set	Test Set
R^2^	0.933	0.869	0.993	0.885	0.993	0.893	0.993	0.908
MAE	2.726	5.249	0.330	4.595	0.367	4.201	0.238	4.057
RMSE	3.576	6.501	1.185	6.088	1.192	5.855	1.172	5.446

**Table 5:** The constraint range and constraint condition of the mix proportion of SCBA concrete.

Constraint Item	Constraint Range	Constraint Condition
the relative mass replacement rate of SCBA (*R*_SCBA_/%)	0~35	0 ≤ *R*_SCBA_ ≤ 35
cement content (*C*/kg/m^3^)	280~500	280 ≤ *C* ≤ 500
water–cement ratio (*w*/*c*)	0.3~0.55	0.3 ≤ *w*/*c* ≤ 0.55
fine aggregate (*FA*/kg/m^3^)	600~900	600 ≤ *FA* ≤ 900
coarse aggregate (*CA*/kg/m^3^)	700~1300	700 ≤ *CA* ≤ 1300
the compressive strength of SCBA concrete (*f*_c_/MPa)	C30~C50	$f_{cu,0}\geq f_{cu,k}+1.645\sigma$

**Table 6:** NSGA-III parameter setting.

Parameter	Population Size	Maximum Generations	Mutation Probability	Crossover Probability	Offspring Population Size
Value	100	50	0.2	0.7	50

**Table 7:** The optimal solution of NSGA-III and the corresponding solution set.

Mixture	SCBA Concrete Mix					Target Performance		
	SCBA Replacement Ratio (%)	Cement Content (kg/m^3^)	Water-to-Cement Ratio	Fine Aggregate Content (kg/m^3^)	Coarse Aggregate Content (kg/m^3^)	Predicted Compressive Strength (MPa)	CO_2_ Emission (kg/m^3^)	Cost (yuan)
1	2.65	330.22	0.42	771.01	943.27	54.86	277.99	389.31
2	4.57	319.47	0.45	772.96	745.58	49.71	269.08	422.59
3	7.31	282.36	0.31	867.05	887.48	44.10	238.72	498.29

**Table 8:** Experimental results of the optimal solution set.

Test Piece	SCBA Concrete Mix					Target Performance		
	SCBA Replacement Ratio (%)	Cement Content (kg/m^3^)	Water-to-Cement Ratio	Fine Aggregate Content (kg/m^3^)	Coarse Aggregate Content (kg/m^3^)	Predicted Value (MPa)	Experimental Mean Value (MPa)	Error (%)
1	2.65	330.22	0.42	771.01	943.27	54.86	47.72	14.96
2	4.57	319.47	0.45	772.96	745.58	49.71	44.76	11.06
3	7.31	282.36	0.31	867.05	887.48	44.10	38.54	14.43

## Data Availability

The data presented in this study are openly available in GitHub at https://github.com/fzy01060020/The-data-of-SCBA-concrete-mix-proportions.git (accessed on 20 August 2026), as stated in the manuscript.

## Appendix A

https://github.com/fzy01060020/The-data-of-SCBA-concrete-mix-proportions.git (accessed on 20 August 2026).
